# ^225^Ac-rHDL Nanoparticles: A Potential Agent for Targeted Alpha-Particle Therapy of Tumors Overexpressing SR-BI Proteins

**DOI:** 10.3390/molecules27072156

**Published:** 2022-03-27

**Authors:** Tania Hernández-Jiménez, Guillermina Ferro-Flores, Enrique Morales-Ávila, Keila Isaac-Olivé, Blanca Ocampo-García, Liliana Aranda-Lara, Clara Santos-Cuevas, Myrna Luna-Gutiérrez, Laura De Nardo, Antonio Rosato, Laura Meléndez-Alafort

**Affiliations:** 1 Department of Radioactive Materials, Instituto Nacional de Investigaciones Nucleares, Ocoyoacac 52750, Mexico; tania.hernandez@inin.gob.mx (T.H.-J.); blanca.ocampo@inin.gob.mx (B.O.-G.); clara.cuevas@inin.gob.mx (C.S.-C.); myrna.luna@inin.gob.mx (M.L.-G.); 2 Faculty of Chemistry, Universidad Autónoma del Estado de México, Toluca 50180, Mexico; 3 Faculty of Medicine, Universidad Autónoma del Estado de México, Toluca 50180, Mexico; kisaaco@uaemex.mx (K.I.-O.); larandal@uaemex.mx (L.A.-L.); 4 Department of Physics and Astronomy, University of Padua, 35131 Padova, Italy; laura.denardo@unipd.it; 5 Department of Surgery, Oncology and Gastroenterology, University of Padova, 35138 Padova, Italy; antonio.rosato@unipd.it; 6 Veneto Institute of Oncology IOV-IRCCS, 35138 Padova, Italy; laura.melendezalafort@iov.veneto.it

**Keywords:** scavenger receptor B type I, reconstituted high-density lipoproteins, actinium-225, ^225^Ac-rHDL, targeted alpha-particle therapy

## Abstract

Actinium-225 and other alpha-particle-emitting radionuclides have shown high potential for cancer treatment. Reconstituted high-density lipoproteins (rHDL) specifically recognize the scavenger receptor B type I (SR-BI) overexpressed in several types of cancer cells. Furthermore, after rHDL-SR-BI recognition, the rHDL content is injected into the cell cytoplasm. This research aimed to prepare a targeted ^225^Ac-delivering nanosystem by encapsulating the radionuclide into rHDL nanoparticles. The synthesis of rHDL was performed in two steps using the microfluidic synthesis method for the subsequent encapsulation of ^225^Ac, previously complexed to a lipophilic molecule (^225^Ac-DOTA-benzene-p-SCN, CLog P = 3.42). The nanosystem (13 nm particle size) showed a radiochemical purity higher than 99% and stability in human serum. In vitro studies in HEP-G2 and PC-3 cancer cells (SR-BI positive) demonstrated that ^225^Ac was successfully internalized into the cytoplasm of cells, delivering high radiation doses to cell nuclei (107 Gy to PC-3 and 161 Gy to HEP-G2 nuclei at 24 h), resulting in a significant decrease in cell viability down to 3.22 ± 0.72% for the PC-3 and to 1.79 ± 0.23% for HEP-G2 at 192 h after ^225^Ac-rHDL treatment. After intratumoral ^225^Ac-rHDL administration in mice bearing HEP-G2 tumors, the biokinetic profile showed significant retention of radioactivity in the tumor masses (90.16 ± 2.52% of the injected activity), which generated ablative radiation doses (649 Gy/MBq). The results demonstrated adequate properties of rHDL as a stable carrier for selective deposition of ^225^Ac within cancer cells overexpressing SR-BI. The results obtained in this research justify further preclinical studies, designed to evaluate the therapeutic efficacy of the ^225^Ac-rHDL system for targeted alpha-particle therapy of tumors that overexpress the SR-BI receptor.

## 1. Introduction

High-density lipoproteins (HDLs) are hydrophobic lipid micelles (endogenous nanoparticles; diameter of 5–12 nm) that carry cholesterol esters, free cholesterol, phospholipids, and triglycerides. HDLs transport and release their lipidic content in the liver and into the cytoplasm of different cells by interacting with specific cell membrane proteins. Due to the presence of apolipoprotein AI (Apo AI) as the main HDL peripheral component for targeting the scavenger receptor class B type I (SR-BI), synthetic or reconstituted HDL nanoparticles (rHDL), based on phospholipids and Apo AI, have been used as hydrophobic drug transporters, promoting the research of rHDL as a vehicle for the administration of chemotherapeutic agents [1,2].

The SR-BI is overexpressed on the cell surface of many cancer cells (e.g., ovarian, liver, and prostate cancer) and is a selective and specific receptor for HDL (through Apo AI). The SR-BI–HDL interaction triggers the release of cholesterol from HDL to the cell cytoplasm. Cholesterol is necessary for cell growth and is highly required by cancer cells, because it takes part in the synthesis of new cytoplasmic membranes [2,3].

Radiolabeled rHDLs have been employed for molecular imaging of cancer cells by targeting the SR-BI protein. Perez-Medina et al. attached ^89^Zr-deferoxamine to the ApoE/phospholipids of rHDL; preclinical PET images showed high ^89^Zr-rHDL uptake in tumor-associated macrophages of breast cancer [4]. Recently, preclinical studies of ^99m^Tc-rHDL for SPECT imaging of prostate cancer metastasis have also been reported [5].

Alpha particles are an extraordinary option for targeted radiotherapy due to their capacity to cause damage to cancer cells, because the corresponding deposition of energy at the cellular level is one-hundred times greater than that of β-particles [6]. Targeted alpha-particle therapy (TAT) with ^225^Ac has demonstrated considerable potential in the treatment of advanced prostate cancer [6].

Despite dozens of previous articles on ^225^Ac labeling of metallic nanoparticles, liposomes, and micelles, the preparation of ^225^Ac-HDL nanosystems has not been reported so far. The ^225^Ac is produced primarily from a ^229^Th/^225^Ac generator with subsequent ^225^Ac purification. The ^255^Ac is characterized by decay into multiple alpha-emitting daughter radionuclides (four effective alpha emissions from ^225^Ac, ^221^Fr, ^217^At, and ^213^Po). One possible disadvantage of ^225^Ac-complexes, however, is the recoil energy (~100 keV) of the nucleus during decay, which could induce the breaking of the bond with the chelator or transporter molecule with the consequent release of the daughter radionuclide to healthy tissues [7].

Therefore, the encapsulation of ^225^Ac in rHDL nanoparticles (^225^Ac-rHDL) would prevent the release of daughter radionuclide from the transporter biomolecule due to the recoil energy effect and, at the same time, the specific molecular recognition of ^225^Ac-rHDL by cancer cells that overexpress the SR-BI protein would allow the cytoplasmic internalization of ^225^Ac in tumor cells to produce ablative radiation doses.

This research aimed to prepare ^225^Ac-rHDL and evaluate its preclinical in vitro and in vivo capability as a potential agent for targeted α-therapy of tumors overexpressing SR-BI receptors.

## 2. Results and Discussion

### 2.1. rHDL Assembly and Chemical Characterization

**Figure 1:**
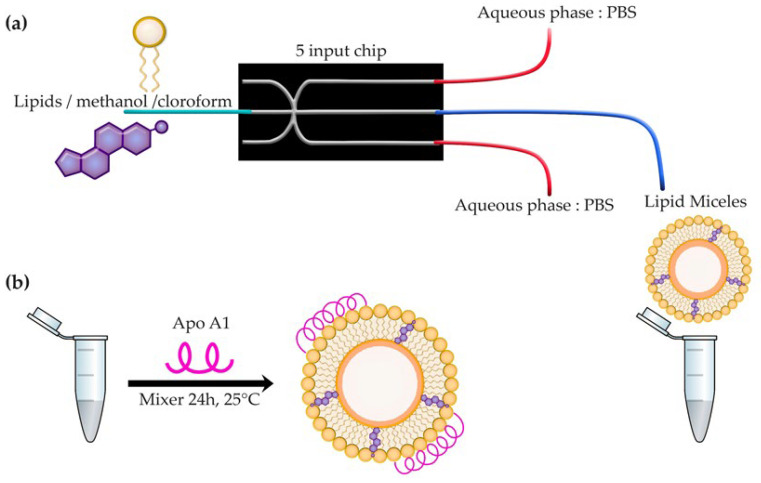
(**a**) Synthesis of lipid micelles. Preparation of micelles via a microfluidic system with hydrodynamic flow focusing, using a 5-way glass 3D chip that allows the diffusion of lipids in water, as well as water in alcohol, until its concentration decreases below the limit of lipid solubility, triggering the formation of lipid micelles. (**b**) rHDL synthesis. In the second step, the incorporation of apolipoprotein Apo AI was performed for the formation of rigid rHDL.

**Figure 2:**
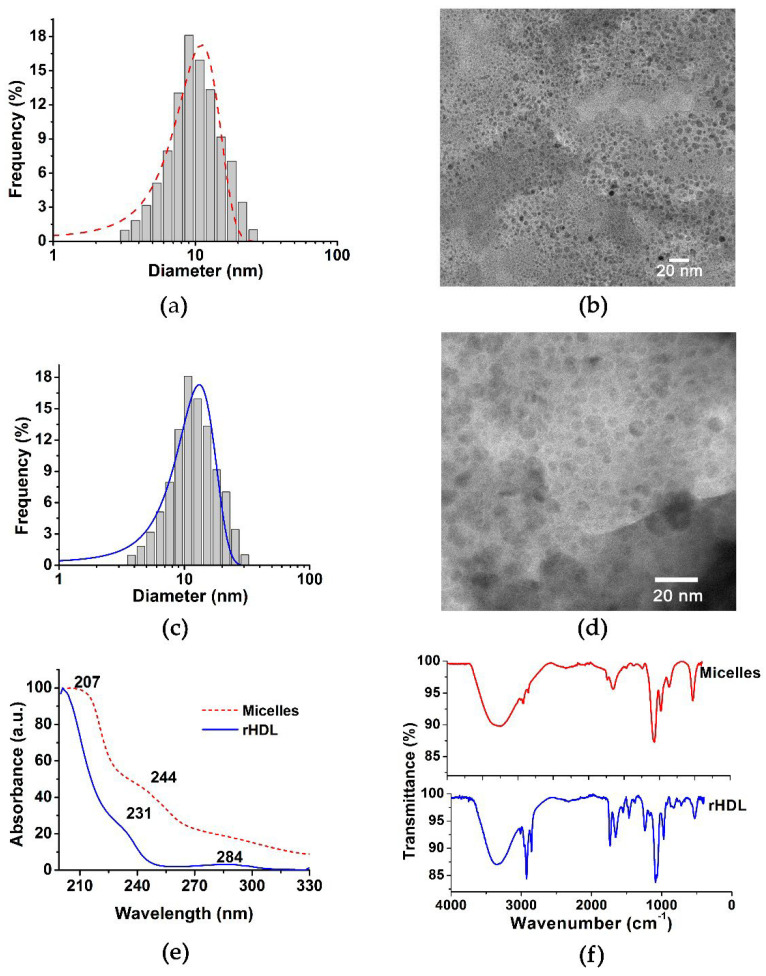
(**a**) Size distribution of lipid micelles by DLS, (**b**) TEM micrographs of lipid micelles, (**c**) size distribution of rHDL by DLS, (**d**) TEM micrographs of rHDL, (**e**) UV-Vis spectra of lipid micelles (red) and rHDL (blue), and (**f**) FT-IR spectra of lipid micelles (red) and rHDL (blue).

rHDL nanoparticles, generated from lipid micelles assembled in a microfluidic system, followed by the incorporation of Apo AI (Figure 1), were obtained with monomodal and monodisperse size distributions, with a hydrodynamic diameter of 13.14 ± 0.20 nm and a polydispersity index of 0.176, as determined by dynamic light scattering (DLS) (Table 1). In addition, transmission electron microscopy (TEM) micrographs demonstrated the presence of nanoparticles with a mean diameter of 11.10 ± 0.17 nm, spherical shape, and a homogeneous and uniform distribution (Table 1 and Figure 2). The protein content analysis of rHDL indicated an Apo AI concentration of 0.131 mg/mL.

The FT-IR spectra of the micelles and rHDL are shown in Figure 2. The band at 3400 cm^−1^, observed in the spectrum of lipid micelles and characteristic of the (OH)ʋ groups of the cholesterol molecule, was shifted to 3287 cm^−1^ after Apo AI addition, which is associated with the stretching (OH)ʋ vibration of water molecules remaining in the interface between the lipoprotein and cholesterol, as well as indicative of the Apo AI incorporation to the lipid micelles due to interactions of the lysine primary amine residues with the lipid groups [8,9]. The band of highest intensity, observed at 2955 cm^−1^, corresponds to an asymmetric stretch (CH)ʋ, characteristic of the methyl groups present in the phospholipid chains, while the band at 2854 cm^−1^ corresponds to the symmetric stretch (CH)ʋ of the methylene groups that constitute the rHDL. However, in the spectrum obtained from the lipid micelles, these signals may be superimposed by the broad band of the vibration (OH)ʋ. The region from 1500 cm^−1^ to 1800 cm^−1^ is characteristic of (C=O)ʋ vibrations of the ester bonds present in the phospholipid chains. At 1650 cm^−1^, there is an important vibration in the rHDL spectrum, which corresponds to Amide I of the α-helix of Apo AI. At 1538 cm^−1^, a band attributed to amide II corresponding to (N-H)ʋ bending and (C-N)ʋ stretching was observed [8,9].

In the case of lipid micelles, a wide band of lower intensity at 1674 cm^−1^ was attributed to the (C=C)ʋ vibration characteristic of the second ring of cholesterol. The broad band at 1400 cm^−1^, in the rHDL spectrum, corresponds to the asymmetric stretching of the (COO-)ʋ groups of the aspartate and glutamate residues, which are believed to be involved in the coupling of lipoproteins [10].

In summary, the spectrum of lipid micelles contains characteristic bands attributable to the cholesterol and free cholesterol esters as components of the mixture for the formation of micelles. In the rHDL spectrum, the Apo AI association with acidic lipid membranes, through interactions between lysine residues and negatively-charged lipid groups, can be appreciated. Interactions with the anionic phospholipids increase the Apo AI α-helix content, which is also an important factor in recognizing the specific molecular target [10].

The UV-Vis spectrum of the micelles showed a wide absorption band at 207 nm and a shoulder at 244 nm (Figure 2), in agreement with those reported for cholesterol compounds [8]. The band at 244 nm is associated with the π → π * transitions of the α and β unsaturated ketones present in the cholesterol structure. After incorporating Apo AI into the micelles, an absorption band at 284 nm was observed, which indicates the coupling of proteins to lipid micelles. A slight band at 231 nm was also observed in the spectrum of rHDL, which is associated with the lipid–protein interaction.

### 2.2. The 225Ac-rHDL

**Figure 3:**
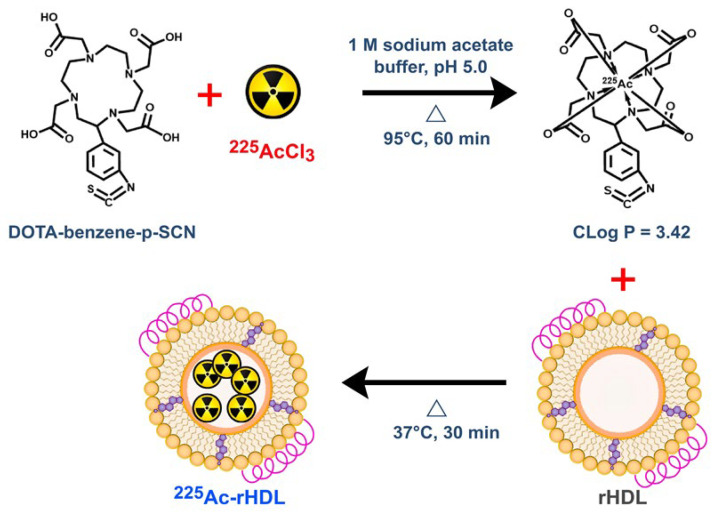
Schematic steps of the incorporation of ^225^Ac into rHDL nanocapsules (^225^Ac-rHDL).

The incorporation of ^225^Ac was conducted efficiently with the rHDL vesicles by passive internalization through the encapsulation of ^225^Ac previously complexed to a lipophilic molecule (^225^Ac-DOTA-benzene-p-SCN) with a CLog P of 3.42 (Figure 3). As a result, the ^225^Ac-rHDL system was obtained with a labeling efficiency of 85 ± 3% and a radiochemical purity greater than 99%, as determined by ultrafiltration.

### 2.3. In Vitro Studies

#### 2.3.1. Serum Stability of the ^225^Ac-rHDL System

The ^225^Ac-rHDL was incubated in fresh human serum at 37 °C for 10 d. During the analysis of the samples at 24, 72, 144, and 240 h, the radioactivity associated with the rHDL system remained greater than 99%. It is important to mention that, according to what has previously been reported [11,12], the ^225^Ac daughters (alpha emitters) can escape from the carrier vehicle and irradiate non-target tissues. However, the successful ^225^Ac-DOTA-benzene-p-SCN encapsulation into the hydrophobic vesicle of rHDL allowed the retention of ^225^Ac and its progeny within the nanosystem.

#### 2.3.2. Cell Viability Assay and Dose to the Nucleus

Cell viability of PC-3 and HEP-G2 cells (SR-BI positive), and fibroblasts (negative control), was evaluated after treatment with a) ^225^Ac-DOTA-benzene-p-SCN (control) and b) ^225^Ac-rHDL at 37 °C for 1 h.

**Figure 4:**
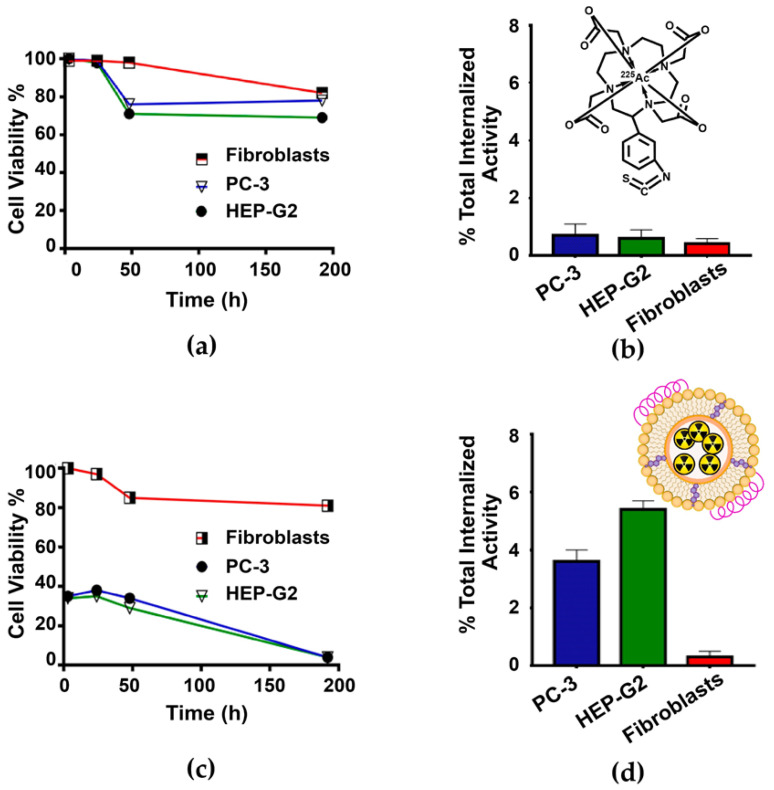
Cell viability assay: (**a**) PC-3, HEP-G2, and fibroblast cell lines treated with the ^225^Ac-DOTA-benzene-p-SCN system (control); note that viability above 80% was maintained after 192 h for all cell lines. (**b**) Internalization of ^225^Ac-DOTA-benzene-p-SCN in PC-3, HEP-G2 cells, and fibroblasts; note that the low cellular internalization of the ^225^Ac-DOTA-benzene-p-SCN complex (without significant difference among cells, *p* > 0.05, two-way ANOVA) correlates with a relatively low effect on cell viability at 192 h. (**c**) PC-3, HEP-G2, and fibroblast cell lines treated with the ^225^Ac-rHDL system; note a viability of 62–64% after 24 h and less than 3.5% at 192 h for the PC-3 and HEP-G2 cell lines, which overexpress the SR-BI protein. (**d**) Internalization of ^225^Ac-rHDL in PC-3, HEP-G2 cells, and fibroblasts; note a significant cellular internalization of the ^225^Ac rHDL complex in PC-3 and HEP-G2 cells, which correlates with a significant effect on cell viability.

The cell viability of PC-3 and HEP-G2 treated with ^225^Ac-DOTA-benzene-p-SCN was not significantly different between them nor with regard to that of fibroblasts (*p* > 0.05, two-way ANOVA) (Figure 4a). Furthermore, a low ^225^Ac-DOTA-benzene-p-SCN internalization was observed in all cell lines (Figure 4b). These results indicate that the ^225^Ac-DOTA-benzene-p-SCN system, despite being a hydrophobic compound, does not have an interaction mechanism with the surface of the cell membrane that would allow ^225^Ac to be internalized into the cell cytoplasm. In contrast, the PC-3 and HEP-G2 cells that received treatment with the ^225^Ac-rHDL nanosystem showed cell viability of 54.67± 3.16% and 53.12 ± 2.93% at 3 h, respectively, with a slight increase at 24 h (62.08 ± 2.44% for PC-3 and 64.32 ± 3.38% HEP-G2) (Figure 4c). A possible explanation could be that cell repair mechanisms are stimulated upon receiving an initial dose of radiation, promoting cell proliferation in both cell lines [13,14]. Nevertheless, cell viability decreased to 3.22 ± 0.72% for the PC-3 cell line at 192 h after ^225^Ac-rHDL treatment and to 1.79 ± 0.23% for the HEP-G2 cell line, as a result of the significant internalization of radiation (Figure 4d). In the case of fibroblasts, a cell viability of 81.2 ± 7.21%, and the lowest internalization index regarding the two cell lines (PC-3 and HEP-G2), was observed after ^225^Ac-rHDL treatment (Figure 4d). As is known, fibroblasts poorly express the SR-BI receptor, and when they interact with rHDL, a different mechanism with slight and unspecific internalization can also occur [15,16,17].

The calculation of the absorbed dose, from the cytoplasm to the nucleus of the different cell lines at different times after treatment with the ^225^Ac-rHDL system, was also evaluated (Table 2). The value used as the dose factor (DF) for the dose calculations was ${\left(DF^{\alpha }+DF^{e,ph}\;\right)}_{Ac-225\left(n\leftarrow Cy\right)}=$8.96 × 10^−2^ Gy/Bq·s, where the four alpha, electron (e), and photon (ph) emissions of the daughters produced by each ^225^Ac nuclear transformation were considered (MIRDcellV2.1 software) [18].

The absorbed radiation dose to the cell nucleus at 192 h after ^225^Ac-rHDL treatment was 1025.5 Gy (256.4 Gy per kBq administered in the well) for HEP-G2 and 682.5 Gy (170.6 Gy per kBq administered in the well) for PC-3, which is 43 and 29 times higher than in the case of fibroblasts, respectively. Therefore, it was demonstrated that the ^225^Ac-rHDL nanosystem is capable of delivering doses of radiation to cause a significant cytotoxic effect, as a result of the ability to internalize ^225^Ac into the cell and the multiple alpha-emitting daughter radionuclides generated inside the cell; that is, the SR-BI receptor, expressed on HEP-G2 and PC-3 cells, interacts with the endogenous rHDL lipoproteins, which induces the deposit of the rHDL content (^225^Ac) directly into the cytoplasm of the cells. An advantage of the alpha radiotherapy system, in addition to its high specificity attributed to rHDL, is that the high number of short-path ionizations, which damage the DNA structure, also inhibit cell repair mechanisms [19,20]. On the other hand, a 20% decrease in cell viability was also observed in fibroblasts with a dose of 8.1 Gy at 48 h after treatment, which can be attributed to the radiosensitivity exhibited by fibroblasts (Figure 4c) [19].

### 2.4. Biodistribution Assay

The biodistribution profile in healthy mice, after intravenous administration of the ^225^Ac-rHDL nanosystem, showed an accumulation of activity, mainly in the liver, with a value of 40.4 ± 2.12% ID, but with a relatively rapid clearance, reaching 6.01 ± 1.07% ID at 192 h (Table 3). Radioactivity in the liver is rapidly removed and excreted due to the liver metabolic dynamics of HDL [21]. Given its nanometric size, ^225^Ac-rHDL elimination occurs both through the hepatobiliary (2.88 ± 0.12% ID at 0.5 h in intestine) and renal (4.85 ± 0.89% ID at 0.5 h in kidney) pathways. Because it is inaccurate to establish the percentage of radioactivity accumulated in the total blood of mice, the % ID in blood was not included in Table 3. However, the activity in blood was 3.91 ± 0.92% ID/g and 1.97 ± 0.81% ID/g at 0.5 h and 24 h, respectively. It should be considered that the elimination time of rHDL in blood plasma is relatively prolonged due to its lipoprotein nature (the half-life in plasma fluctuates between 6 h and 24 h after HDL administration) [21]. Therefore, a prolonged blood circulation of ^225^Ac-rHDL is expected, which could be an advantage in therapeutic applications given that the nanosystem can produce a significant tumor accumulation of radioactivity before radiopharmaceutical elimination [22]. As expected, rHDL was captured by the liver tissue due to its high expression of SR-BI. These results confirm the specificity and passive targeting of SR-BI receptors, as well as their in vivo affinity for rHDL lipoproteins through a natural process of selective uptake and elimination. This behavior is of particular importance when demonstrating that it is possible to minimize long retention of ^225^Ac-rHDL in healthy organs, because, due to the nature of the alpha-emitting daughter radionuclides, high damage to healthy tissues could be produced.

**Figure 5:**
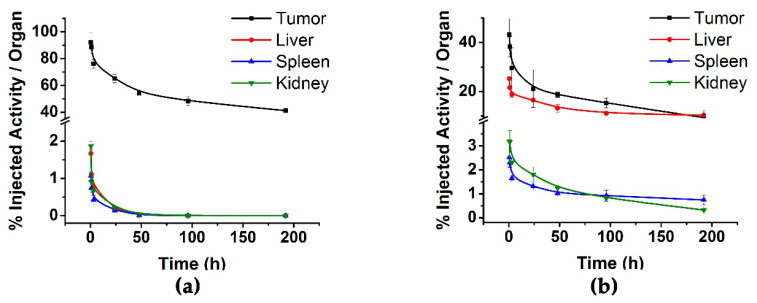
Comparison of the biokinetic profile between the (**a**) ^225^Ac-rHDL nanosystem and (**b**) ^225^Ac-DOTA-benzene-p-SCN in nude mice bearing HEP-G2 tumors after intratumoral administration.

Biodistribution in mice with induced HEP-G2 tumors was performed in order to assess tumor uptake activity as a function of time. The biodistribution profile of ^225^Ac-rHDL, after intratumoral administration, shows that the radioactivity of the system is retained within the tumor masses (Figure 5a), with an uptake of 90.16 ± 2.52% at 0.5 h, with regard to the initially-administered activity. These results confirm the potential of ^225^Ac-rHDL to produce a localized cytotoxic effect. Low ^225^Ac-rHDL accumulation was observed in liver (0.65% ID at 24 h), kidney (0.51% ID at 24 h), and spleen (0.34% ID at 24 h), which indicates that ^225^Ac does not leak in vivo from the rHDL nanocapsule, because unchelated actinium (^225^Ac^3+^) accumulates significantly in the liver [23].

When ^225^Ac-DOTA-benzene-p-SCN was injected intratumorally in nude mice bearing HEP-G2 tumors, a very rapid clearance of radioactivity from the tumor was observed (Figure 5b); and almost 10-fold higher uptake in liver and spleen was seen with regard to the ^225^Ac-rHDL biodistribution pattern (Figure 5b). These findings suggest that although the DOTA chelating agent is highly stable for positive trivalent radiometals [12,24,25], ^225^Ac-DOTA-benzene-p-SCN does not remain in the tumor, because the recoil energy of the ^225^Ac daughters (1000 times greater than the binding energy of any chemical compound) is possibly causing the breaking of chemical bonds, with the consequent retention of ^225^Ac^3+^ and its progeny in the liver as ionic forms [23].

Based on the biodistribution results in healthy and tumor-bearing mice, it is possible to propose ^225^Ac-rHDL as a convenient natural nanocarrier for the use of ^225^Ac in targeted radiotherapy, in a safe and efficient manner.

Table 4 shows the biokinetic models of the ^225^Ac-rHDL nanosystem in tumor and radiation source organs. The results show both the biological or pharmacokinetic model ($q_{h}\left(t\right)$) and the radiopharmacokinetic model ($A_{h}\left(t\right)$), the latter associated with the number of nuclear transformations that occurred in each tissue (N) for the radiation-absorbed dose calculation.

The highest dose occurred in the tumor (649 Gy), while doses were low to the other organs (Table 4), indicating that the energy deposited by ^225^Ac produces ablative radiation doses to the target malignant lesions, avoiding cytotoxic effects on healthy tissues.

As is known, some of the key properties of ^225^Ac as a radionuclide for targeted alpha radiotherapy of micrometastases are: (1) range in tissue of a few cell diameters, (2) high linear energy transfer, which leads to direct damage of the DNA structure (LET = 100 KeV/µm), (3) half-life of 10 days, which allows sufficient time for the administration of the dose and its binding and retention in tumor masses, (4) emission of four alpha particles per nuclear transformation [26,27,28,29]. Another important aspect to consider is that ^225^Ac requires a much lower activity to produce cytotoxic effects at the cellular level with regard to beta emitters, because its energy is deposited in an extremely localized manner. Furthermore, ^225^Ac-rHDL have the appropriate physicochemical properties and size to reach the tumor tissue and locally deposit their content (^225^Ac) within the cytoplasm of tumor cells, being a convenient vehicle for targeted alpha-particle radiotherapy.

**Figure 6:**
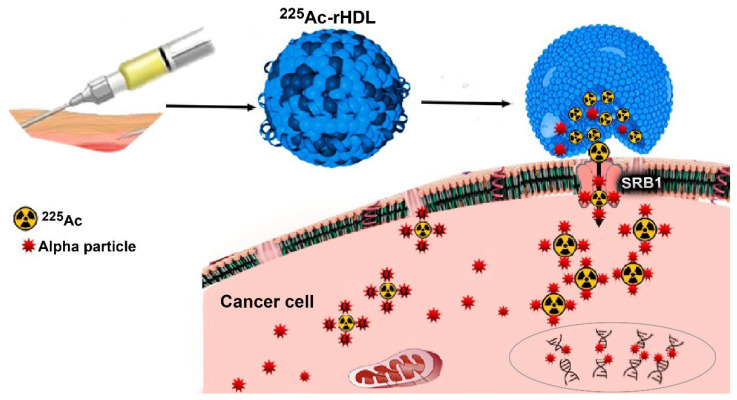
Cellular interaction mechanism of rHDL containing ^225^Ac. Once endogenous rHDL interacts with the SR-BI receptor found on the surface of the cell membrane, its content is released directly into the cell’s cytoplasm. Therefore, ^225^Ac-rHDL is an effective nanosystem for depositing within the malignant cells, an in vivo generator for alpha particle radiotherapy, which delivers lethal radiation doses to cells due to the four alpha particles emitted by ^225^Ac and its progeny in each nuclear transformation. The ionizations produced by ^225^Ac and its daughters produce direct damage to the DNA structure, preventing tumor proliferation.

Although the mechanism by which the interaction of the SR-BI receptor with HDL occurs has not yet been fully elucidated, it is possible that there is a non-aqueous “channel” in the SR-BI receptor that can accommodate cholesterol esters in such a way that it can couple with rHDL and capture its content through an internal tunnel [30] (Figure 6).

The biokinetic profile of the ^225^Ac-rHDL system is comparable to those reported for ^99m^Tc-HYNIC-DA-rHDL, with regard to tumor uptake and clearance because the delivery mechanism is the same [5]. In addition, the accumulation of activity in organs that express SR-BI (mainly liver, spleen, and kidneys) is comparable.

## 3. Materials and Methods

Free cholesterol (FC), egg yolk phosphatidylcholine (EYPC), cholesterol oleate (CE), and sodium cholate reagents were supplied by Landsteiner Scientific, and apolipoprotein Apo AI and human plasma by Alfa Aesar (Thermo Fisher, Tewksbury, MA, USA). Buffer Tris-EDTA was prepared in the laboratory. Dialysis tubbing (14000 Daltons) was obtained from MEMBRA CEL^®^ (Thermo Fisher, Tewksbury, MA, USA). Macrocycle S-2-(4-isothiocyanatobenzyl)-1,4,7,10-tetraazacyclododecane acid, DOTA-benzene-p-SCN (p-SCN-Bn-DOTA) was obtained from Macrocyclics (Dallas, TX, USA). Actinium-225 (^225^Ac), as ^225^AcCl_3_, was supplied from ITM, Germany. The bladder fibroblast cell human cell line, PC-3, and HEP-G2 cell lines (SR-BI positive) were obtained from American Type Culture Collection (ATCC^®^, Manassas, VA, USA).

### 3.1. Preparation of Lipid Micelles and rHDL

The synthesis of the lipid micelles was performed via the microfluidic method with hydrodynamic flow focusing (MFH), using a Dolomite Microfluidics System (Dolomite) equipped with a 150-µm 5-way glass 3D chip, with dimensions of 22.5 mm long, 15 mm wide, and 4 mm high. A 4-way H interface with two 4-way linear connectors and two connector seals were used. Flow patterns in the mixing zone of the microfluidic device were visualized using a high-speed digital microscope (Meros, Dolomite). Fluids were administered by two pressure pumps. The first pump for lipid, ethanol, and chloroform delivery. The second pump for the administration of PBS, with the corresponding flow sensors. The chip used presents a hydrodynamic flow approach that allows the formation of a stable laminar flow confined by two lateral flows.

rHDL was prepared in two steps. In the first, the formation of lipid micelles was performed via the microfluidic method, for which an organic solution consisting of 2 mL of methanol: chloroform (1:0.02 *v*/*v*), containing a mixture of the following lipids, was used: 300 µL of egg yolk phosphatidylcholine (10 mg/mL in methanol), 7 µL of free cholesterol (10 mg/mL in methanol), and 7.5 µL of cholesterol ester (4 mg/mL in chloroform). PBS (pH 7.4) was used as aqueous phase. Both solutions were filtered through a 0.22-µm PVDF membrane before being introduced into the microfluidic device. The organic phase was placed in the central channel and the aqueous solution in the coaxial channels, adjusting the organic phase to a flow of 50 µL/min and the aqueous phase to 500 µL/min, with a total flow ratio (TRF) of 550 µL/min and a flow rate ratio (FRR) of 47. Once the micelles were obtained, the size was measured by dynamic light scattering (DLS) (Nanotrac Wave, Model MN401, Microtract, Montgomeryville, PA, USA). Dialysis for purification (5 °C; 24 h) was performed using a 14000-kDa membrane (MEBRA CEL^®^, Thermo Fisher, Tewksbury, MA, USA) with PBS pH 7.4 to remove any residual solvent. Then, 4 mg/mL of Apo AI and 140 µL of sodium cholate (20 mg/mL) were added. Dialysis was performed again with stirring at 5 °C for 24 h to remove excess surfactant. The obtained solution was filtered using a 0.45-µm Millipore filter and stored at 4 °C.

### 3.2. Physicochemical Characterization of rHDL

#### 3.2.1. Nanoparticle Size

The size of lipid micelles and rHDL was determined via dynamic light scattering (DLS) (Nanotrac Wave, Model MN401, Microtract, Montgomeryville, PA, USA). The size was also determined by transmission electron microscopy (TEM) (JEOL JEM2010 HT microscope operated at 200 kV), with a magnification of 50,000× *g* for micelles and 200,000× *g* for rHDL. Samples for TEM were stained with 2% sodium phosphotungstate solution (pH 7.2) and placed in carbon-lined 200-mesh copper support cells.

#### 3.2.2. Protein Content

The rHDL protein content was determined using a colorimetric assay based on the formation of copper complexes using the BCA (bicinchoninic acid assay).

#### 3.2.3. UV-Vis Spectra

The UV-Vis spectra (Perkin Elmer Lambda Bio spectrometer) of the lipid micelles and rHDL (1 mg/mL) were obtained, in the range of 200 to 500 nm, using a low-volume quartz cell (0.5-mL capacity) and an optical path of 1 cm.

#### 3.2.4. Fourier Transform Infrared Spectroscopy (FT-IR)

The IR spectra of lipid micelles and rHDL were acquired on a PerkinElmer 2000 spectrophotometer with an attenuated total reflection platform (Pike Technologies; Madison, WI, USA). Spectra were acquired from 50 scans at 0.4 cm^−1^, from 400 to 4000 cm^−1^.

### 3.3. Preparation of ^225^Ac-DOTA-Benzene-p-SCN

DOTA-benzene-p-SCN (pDOTA-Bz-SCN) (1 mg) was dissolved in 50 µL of 0.01 M NaOH, adjusting to a final volume of 2 mL with 1 M acetate buffer (pH 5.0). The ^225^Ac progeny in the decay chain have γ emissions. Therefore, to quantify the ^225^Ac activity, under secular equilibrium, a CRC-55tR radioisotope calibrator setting was used (Capintec Inc., Mirion Technologies, Florham Park, NJ, USA) (calibration # 775 with a 5× multiplier), which mainly considers the 213Bi 440 keV γ emission [12,31]. To 200 µL of the DOTA-benzene-p-SCN, 50 µL of ^225^AcCl_3_ (18 MBq in 0.01 M HCl) were added. Finally, the mixture was incubated at 95 °C for 30 min. The radiochemical purity of ^225^Ac-DOTA-benzene-p-SCN was determined using an HPLC reverse phase chromatography system (Waters Corporation, Milford, MA, USA). The separation of the samples was performed with a Waters µBondapak-C18 column at a flow rate of 1 mL/min. A linear gradient of H_2_O with 0.1% TFA (A)/CH_3_CN with 0.1% TFA (B), from 100% to 10% of A in 20 min, was used. Fractions of 0.5 mL (40 fractions) were collected and the activity measured in a well-type scintillation NaI(Tl) detector (Auto In-v-tron 4010; NML Inc., Houston, TX, USA). The retention times of ^225^AcCl_3_ and ^225^Ac-DOTA-benzene-p-SCN were 3 min and 12.5 min, respectively. The ^225^Ac-DOTA-benzene-p-SCN was obtained with radiochemical purity greater than 99%.

### 3.4. Preparation of ^225^Ac-rHDL

To 1 mL of the previously prepared rHDL, 200 µL of ^225^Ac-DOTA-benzene-p-SCN (12 MBq) were added, and the mixture was incubated at 37 °C for 1 h to obtain the ^225^Ac-rHDL nanosystem.

The labeling efficiency of ^225^Ac-rHDL was evaluated by ultracentrifugation (2500 g for 0.5 h; MWCO 30-kDa filter units, Amicon Ultra, Millipore, MilliporeSigma, Burlington, MA, USA). The fraction that is not retained in the membrane was considered as the radioactivity associated with ^225^Ac-DOTA-benzene-p-SCN and ^225^Ac^3+^ that was not bound to rHDL, while the fraction that remained in the membrane represents the ^225^Ac-rHDL system. Both fractions were counted in a well-type scintillation NaI(Tl) detector to evaluate the labeling efficiency. The radioactive sample that remained in the filter membrane was resuspended in 10 mL of acetate buffer (pH 5.0)/0.1% ascorbic acid. Finally, a sample of the final solution (100 µL; activity measured in a well-type scintillation NaI(Tl) detector) was centrifuged again under the same conditions (2500× *g* for 0.5 h; MWCO 30-kDa filter units) to verify the radiochemical purity (RP) (RP = (activity remained in the filter membrane/total activity) × 100).

### 3.5. Serum Stability of the ^225^Ac-rHDL System

The ^225^Ac-rHDL system (150 µL) was added to 5 mL of diluted human serum (5×). The solutions (*n* = 3) were incubated at 37 °C for 10 days. A total of 1 mL of sample was taken at 24, 72, 144, and 240 h to evaluate the stability of the nanosystem. To each sample, 300 µL of TFA was added for protein precipitation. Samples were centrifuged at 1200× *g* for 5 min. The activity (measured in a in a well-type scintillation NaI(Tl) detector) obtained in the sediment corresponded to ^225^Ac-rHDL, and the activity in the supernatant, to the free fraction of ^225^Ac^3+^ leaked from the rHDL nanosystem.

### 3.6. Cell Internalization

The HEP-G2 (human hepatocellular carcinoma), PC-3 (human prostate cancer), and fibroblasts cell lines, used for the internalization assays, were cultured in RPMI-1640 medium containing antibiotics (penicillin and streptomycin; 100 µg/mL) and fetal bovine serum at a concentration of 15% in an atmosphere of 5% carbon dioxide and 37 °C.

HEP-G2, PC-3, and fibroblast cells were harvested and diluted in PBS (pH 7.4). Each cell line (1 × 10^5^ cells/tube) received two different treatments: (a) ^225^Ac-rHDL (4 kBq/200 µL PBS at pH 7.4) (*n* = 3), and (b) ^225^Ac-DOTA-benzene-p-SCN (4 kBq/200 µL PBS at pH 7.4) (*n* = 3). Cells were incubated with each treatment at 37 °C for 1 h. After the incubation time, tubes were measured in a well-type NaI(Tl) scintillation detector to determine the initial activity (100%). The tubes were centrifuged at 500× *g* for 10 min. The button was washed 2 times with PBS, pH 7.4. Then, a mixture of acetic acid/0.5 M NaCl was added, and the tubes were centrifuged again at 500× *g* for 10 min. The supernatant was removed, and activity of the button was measured, which corresponded to the percentage of activity internalized in the cells with regard to the initial activity.

### 3.7. Cell Viability Assay

The cytotoxic effect on HEP-G2, PC-3, and fibroblast cells after treatment with (a) ^225^Ac-rHDL and (b) ^225^Ac-DOTA-benzene-p-SCN was evaluated by performing an assay of mitochondrial dehydrogenase activity using the XTT kit (Roche Holding AG, Rotkreuz, Switzerland). Briefly, HEP-G2, PC-3, and fibroblast cells (1 × 10^4^ cells/well) were seeded in 96-well microtiter plates. The medium was removed after overnight incubation and the cells were incubated for 1 h with each treatment (4 kBq/200 µL). After removing the treatments, the cells were kept at 37 °C, 5% carbon dioxide, and 85% relative humidity. Cell viability was assessed at 3, 24, 48, and 192 h by spectrophotometric measurements (microplate absorbance reader, EpochTM, BioTek) at 450 nm. Fibroblast cells that did not receive treatment were considered as the control group with 100% cell viability at the different time points.

### 3.8. Radiation-Absorbed Dose Calculation

For the calculation of the total number of nuclear transformations (N)(Bq^.^s) that occurred in the cytoplasm of the PC-3, HEP-G2, and fibroblast cells at different times, the mathematical integration of the activity (^225^Ac) in the cytoplasm ($A_{h}$), as a function of time ($N=\int_{t=1}^{t=2}A_{h}\left(t\right)dt$), was performed. The absorbed dose was calculated by multiplying N by the value of the dose factor (DF)(Gy/Bq^.^s). The DF value (from the cytoplasm to the nucleus) was calculated by using the MIRDcellV2.1 software, considering cells with a diameter of 20 µm, a nucleus radius of 3 µm, and a density of 1 mg/mL. For the dose calculation, the progeny of ^225^Ac with significant decay yield (^225^Ac, ^221^Fr, ^217^At, ^213^Bi, ^213^Po, ^209^Tl, ^209^Pb) was considered, as expressed in Equation (1):

(1) $${\bar{D}}_{225Ac-rHDL\;\left(n\leftarrow Cy\right)}=\underset{j=1}{\sum }N_{\left(Cy\right)}\underset{k}{\sum }b_{k}DF_{k}^{\alpha }{}_{\left(n\leftarrow Cy\right)}+\underset{j=1}{\sum }N_{\left(Cy\right)}\underset{k}{\sum }b_{k}DF_{k}^{e,\;ph}{}_{\left(n\leftarrow Cy\right)}$$

${\bar{D}}_{225Ac-rHDL\;\left(n\leftarrow Cy\right)}$ = radiation-absorbed dose (Gy) to the cell nucleus.
$b_{k}$ = branching fraction for daughter *k.*
$DF_{k}^{\alpha }{}_{\left(n\leftarrow Cy\right)}$ = absorbed dose to the cell nucleus from α emission per nuclear transformation (Gy/Bq·s) from decay of daughter radionuclide “k” originated in the cytoplasm.
$DF_{k}^{e,\;ph}$ = absorbed dose to the cell nucleus from electron (e) and photon (ph) emission per nuclear transformation (Gy/Bq·s) from decay of daughter radionuclide “k” originated in the cytoplasm.
$N_{\left(Cy\right)}$ = total number of nuclear transformations from each daughter radionuclide “k” in the cytoplasm.

### 3.9. Biodistribution Studies

A total of sixty-three animals were injected in three groups: one group of healthy mice (*n* = 21), and two groups of mice bearing HEP-G2 tumors (*n* = 42). In vivo studies with Balb-C mice (7-week-old; weight of 18–20 g) were performed in accordance with the corresponding ethical regulations for handling laboratory animals (“Official Mexican Standard” NOM-062-ZOO-1999). Mice were injected with the ^225^Ac-rHDL system (50 µL, 0.9 MBq) in the tail vein and sacrificed at 0.5, 1, 3, 24, 48, 96, and 192 h (*n* = 3). The spleen, liver, kidneys, lungs, intestine, and heart were removed to measure their activity in a (NaI(Tl)) radioactivity counter, to determine the percentage of injected dose (% ID) per organ, with regard to the total injected activity.

Nude mice bearing HEP-G2 tumors (0.22 ± 0.03 g) were injected intratumorally with the ^225^Ac-rHDL system (50 µL, 1.0 MBq) and sacrificed at 0.5, 1, 3, 24, 48, 96, and 192 h (*n* = 3). As a control, the ^225^Ac-DOTA-benzene-p-SCN system (50 µL, 1.0 MBq) (*n* = 3) was administered and mice were sacrificed at the same time. In all mice, the tumor, liver, spleen, and kidney were removed for activity measurement in a (NaI(Tl)) radioactivity counter, to determine the percentage of injected dose (% ID) per organ, with regard to the total injected activity.

The values obtained from % ID of each organ or tumor were adjusted to exponential functions to obtain the biokinetic models $(q_{h}\left(t\right)$). The $A_{h}\left(t\right)$ functions were obtained by decay-correcting the biokinetic models; that is, by adding to the biological constant $(\lambda_{B}$) the radioactive constant $(\lambda_{R}$), as follows (Equation (2)):

(2) $$A\left(t\right)\;=Be^{-\left(\lambda_{R}+\lambda_{B}\right)t}+Ce^{-\left(\lambda_{R}+\lambda_{B}\right)t}+De^{-\left(\lambda_{R}+\lambda_{B}\right)t}$$

To obtain the biokinetic model, each of the radionuclides generated in the ^225^Ac decay chain was considered as described above. The total number of nuclear transformations (N) in each murine organ were obtained by the mathematical integration (from t = 0 to t = ∞) of the biokinetic models. The DF values were obtained via the OLINDA 2.0 software, considering a normalized tumor mass of 1.0 g and all the progeny of ^225^Ac.

## 4. Conclusions

In this study, the ^225^Ac-rHDL system was prepared and evaluated as a potential targeted radiotherapeutic agent. The results showed adequate physicochemical properties of the rHDL nanocarrier to specifically deposit ^225^Ac into the cytoplasm of HEP-G2 and PC-3 cancer cells and produce a significant cell cytotoxic effect. Biodistribution studies of ^225^Ac-rHDL in healthy mice showed mainly liver uptake with hepatobiliary and renal excretion without appreciable accumulation in other tissues, while, in tumor-bearing mice, the ^225^Ac-rHDL nanosystem remained stable in the tumors and generated ablative radiation doses. The results obtained in this research justify further preclinical studies designed to evaluate the therapeutic efficacy of ^225^Ac-rHDL for targeted alpha-particle therapy of tumors that overexpress the SR-BI receptor.

## Figures and Tables

**Table 1:** Physical characteristics of micelles and rHDL nanoparticles.

Parameter	Lipid Micelles	rHDL
Particle diameter (nm), DLS	11.03 ± 0.04	13.14 ± 0.20
Particle diameter (nm), TEM	10.20 ± 0.13	11.10 ± 0.17
Polydispersity index	0.121	0.176
Protein concentration (mg/mL)	NA ^1^	0.131

^1^ Not applicable.

**Table 2:** Mean radiation-absorbed dose (*Gy*) from the cytoplasm (*Cy*) to the cell nucleus (*n*), considering the internalized activity (Bq/cell) with respect to the total initial activity administered as treatment of ^225^Ac-rHDL (4.0 kBq/well) (1 × 10^4^ cells/well) for each cell line.

Time (h)	Fibroblast	PC-3	HEP-G2
	Radiation Dose $\left(n\leftarrow Cy\right)$	Radiation Dose $\left(n\leftarrow Cy\right)$	Radiation Dose $\left(n\leftarrow Cy\right)$
3	0.5	14.0	20.7
24	3.9	107.3	161.4
48	8.1	208.2	312.2
192	23.8	682.5	1025.5
Internalized Activity (Bq/cell)	0.0005	0.0144	0.0216

**Table 3:** Biodistribution of the ^225^Ac-rHDL nanosystem in healthy mice (Balb-c) after intravenous injection. The percentage of the injected dose per organ (% ID), at various times, is shown (mean ± SD, *n* = 3).

Tissue	Time (h)						
	0.5	1	3	24	48	96	192
Liver	40.40 ± 2.12	31.87 ± 3.19	23.68 ± 1.13	18.27 ± 2.32	11.12 ± 2.21	8.42 ± 1.32	6.01 ± 1.07
Spleen	1.10 ± 0.20	1.30 ± 0.10	1.74 ± 0.24	1.61 ± 0.21	1.02 ± 0.09	0.92 ± 0.15	0.74 ± 0.08
Lung	1.02 ± 0.32	0.87 ± 0.21	0.54 ± 0.11	0.47 ± 0.10	0.21 ± 0.06	0.11 ± 0.08	0.04 ± 0.01
Kidney	4.85 ± 0.89	3.14 ± 0.47	2.63 ± 0.23	1.98 ± 0.82	1.23 ± 0.45	0.81 ± 0.28	0.32 ± 0.12
Intestine	2.88 ± 0.12	1.77 ± 0.92	1.25 ± 0.15	0.84 ± 0.31	0.81 ± 0.12	0.68 ± 0.10	0.42 ± 0.06
Heart	0.50 ± 0.14	0.34 ± 0.09	0.21 ± 0.05	0.17 ± 0.04	0.09 ± 0.02	0.05 ± 0.02	0.01 ± 0.01

**Table 4:** Biokinetic models and average radiation-absorbed doses in different tissues (liver, kidney, spleen, and HEP-G2 tumor) of mice intratumorally injected with ^225^Ac-rHDL (1 MBq).

Organ	Biokinetic Model	$\int_{0}^{\infty }q_{h}\left(t\right)dt$ Biological Residence Time (h)	$N=\int_{0}^{\infty }A_{h}\left(t\right)dt$ Total Nuclear Transformations	Absorbed Dose (Gy)
Tumor	$q_{h}\left(t\right)=26.7e^{-0.542t}+46.4e^{-0.0088t}+26.2e^{-0.000039t}$ $A_{h}\left(t\right)=26.7e^{-0.5449t}+46.4e^{-0.0117t}+26.2e^{-0.0029t}$	6772	130	649.20
Liver	$q_{h}\left(t\right)=0.979e^{-0.0784t}+0.929e^{-1.66t}+3.71e^{-4.88t}$ $A_{h}\left(t\right)=0.979e^{-0.0813t}+0.929e^{-1.6629t}+3.71e^{-4.8829t}$	0.138	0.928	2.17
Kidneys	$q_{h}\left(t\right)=0.619e^{-0.0583t}+0.193e^{-0.0582t}+8.68e^{-4.16t}$ $A_{h}\left(t\right)=0.619e^{-0.0612t}+0.193e^{-0.0611t}+8.68e^{-4.1629t}$	0.160	0.153	2.06
Spleen	$q_{h}\left(t\right)=0.484e^{-0.0588t}+1.27e^{-1.51t}+0.884e^{-13.6t}$ $A_{h}\left(t\right)=0.484e^{-0.0617t}+1.27e^{-1.5129t}+0.884e^{-13.6029t}$	0.091	0.087	3.18

## Data Availability

Not applicable.

## Sample Availability

Samples of the compounds not available are available from the authors.
